# Genetic diversity and population structure of the *Dermacentor nuttalli* in Northern China inferred from microsatellite markers

**DOI:** 10.1186/s13071-026-07426-w

**Published:** 2026-05-07

**Authors:** Chunfu Li, Mengyun Liu, Saira Afzal, Muhammad Uzair Mukhtar, Nazrullozoda Sulaimon, Eman E. El Shanawany, Bolor Bold, Chimedtseren Bayasgalan, Rui Ma, Shurong Wang, Jinghui Zhao, Zhebin Hu, Benguang Zhang, Zhaoan Sheng, Lijuan Liu, Zengyun Hu, Zihan Zhao, Jian Li, Fang Luo, Wei Hu, Xinyu Feng

**Affiliations:** 1 School of Life Sciences, Inner Mongolia University, Hohhot, 010070 China; 2 Department of Medical Entomology and Parasitology, Institute of Public Health, Lahore, 54000 Pakistan; 3 Institute of Veterinary Medicine of the Tajik Academy of Agricultural Sciences, 734025 Dushanbe, Republic of Tajikistan; 4 Department of Parasitology and Animal Diseases, National Research Centre, El Buhouth St., Dokki, Cairo, 12622 Egypt; 5 National Center for Zoonotic Diseases, Songinokhairkhan District, Ulaanbaatar, 18131 Mongolia; 6 Department of Infectious Diseases and Microbiology, School of Veterinary Medicine, Mongolian University of Life Sciences, Ulaanbaatar, 17024 Mongolia; 7 Shandong Institute of Parasitic Diseases, Shandong First Medical University–Shandong Academy of Medical Sciences, Jining, 271016 China; 8 School of Public Health, Shandong First Medical University–Shandong Academy of Medical Sciences, Jinan, 271016 China; 9 Department of Pathogenic Biology, Jining Medical University, Jining, 272067 China; 10 School of Global Health, Chinese Center for Tropical Diseases Research, Shanghai Jiao Tong University School of Medicine, Shanghai, 20025 China; 11 Shanghai Jiao Tong University–The University of Edinburgh One Health Center, Shanghai, 20025 China; 12 Inner Mongolia Helan Mountain National Nature Protection Bureau, Alxa League, 750306 China; 13 Basic Medical College, Guangxi University of Chinese Medicine, Nanning, 530001 China; 14 Ministry of Education Key Laboratory for Biodiversity Science and Ecological Engineering, School of Life Sciences, Fudan University, Shanghai, 200438 China; 15 State Key Laboratory of Genetic Engineering, School of Life Sciences, Fudan University, Shanghai, China

**Keywords:** *Dermacentornuttalli*, Microsatellite, Genetic diversity, Population structure

## Abstract

**Background:**

*Dermacentor nuttalli* is a dominant tick species in northern China and adjacent regions, where it poses significant threats to public health and the livestock industry through the transmission of diverse zoonotic pathogens. Understanding its population genetic structure is crucial for elucidating dispersal patterns and informing control strategies. However, the lack of high-resolution molecular markers has hindered such investigations. This study aimed to develop microsatellite markers and use them to assess the genetic diversity and structure of *D. nuttalli* populations across northern China.

**Methods:**

Genome-wide microsatellite mining was conducted using th*e D. nuttalli* reference genome. A total of 192 adult ticks were collected from five geographic locations in northern China. Genotyping was performed using 15 novel, highly polymorphic markers selected through a multi-step filtering process. Genetic diversity, population structure, molecular variance, gene flow and isolation by distance were investigated in population genetic analyses.

**Results:**

The 15 selected markers exhibited high polymorphism, with a mean polymorphic information content of 0.66. Genetic diversity varied among populations, with the Hulunbuir population showing the highest diversity levels. Population structure analyses consistently revealed two primary genetic clusters: one comprising populations from Yan’an, Bayannur, Ulanqab and Hulunbuir, and another consisting solely of the Hohhot population. Analysis of molecular variance indicated that 36.9% of the total genetic variation occurred among populations, reflecting substantial genetic differentiation (F_ST_ [fixation index] = 0.37). While gene flow was evident among some populations, it was limited between the two identified clusters. Notably, no significant isolation-by-distance pattern was detected (*P* > 0.05).

**Conclusions:**

This study provides the first set of microsatellite markers for *D. nuttalli* and reveals a complex population structure in northern China. Host movement and anthropogenic factors appear to shape this structure more strongly than geographic distance. Notably, the isolated Hohhot population parasitizing wild hedgehogs suggests host-associated genetic differentiation and relatively localized transmission dynamics. These findings highlight the need for differentiated control strategies, including wildlife-focused surveillance in peri-urban areas and targeted livestock management in pastoral systems.

**Graphical Abstract:**

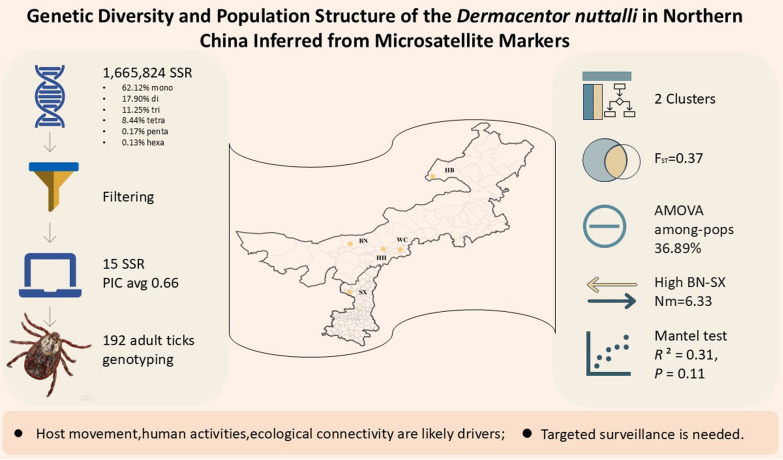

**Supplementary Information:**

The online version contains supplementary material available at 10.1186/s13071-026-07426-w.

## Background

*Dermacentor nuttalli* is a dominant tick species in arid steppe ecosystems and is widely distributed across southern Russia, Mongolia and northern China [1]. This tick primarily parasitizes large herbivorous livestock such as cattle and sheep, as well as wild mammals. It completes its life-cycle through blood-feeding, during which it transmits various pathogens, including tick-borne encephalitis virus, *Rickettsia*, *Coxiella burnetii*, *Anaplasma* and *Borrelia* [2–7]. In recent years, factors such as climate change, shifts in host range and intensified human activities have significantly altered the suitable habitats and host networks of *D. nuttalli* [8]. Such ecological disturbances may not only cause fluctuations in population abundance and shifts in geographical distribution boundaries, but also shape population genetic structure and evolutionary trajectories by influencing isolation and gene flow [9]. Although recent advances in metagenomics and ecological niche modeling have characterized the pathogen profiles and predicted the shifting habitat suitability of *D. nuttalli*, critical knowledge gaps remain [8]. Specifically, the actual genetic connectivity among regional populations and the precise host-mediated dispersal routes driving its expansion are still largely unresolved. Therefore, systematic analyses of the genetic diversity and structure of *D. nuttalli* populations are crucial for improved understanding of its dispersal mechanisms and formulating scientific control strategies.

In recent years, population genetic studies of ticks have gradually transitioned from traditional molecular markers to genome-wide approaches. Early investigations primarily relied on sequence-based markers from specific gene regions, such as mitochondrial DNA (mtDNA) and the internal transcribed spacer (ITS) of ribosomal DNA [10, 11], as well as locus-specific polymorphism markers, including simple sequence repeats (SSRs) and amplified fragment length polymorphisms (AFLPs) [12, 13]. These approaches contributed substantially to current understanding of broad-scale phylogeographic patterns and population structure. However, locus-specific markers may lack sufficient resolution to resolve subtle genetic differentiation and dynamic evolutionary relationships among closely related populations. For example, mitochondrial genomes, due to maternal inheritance, are susceptible to introgression and incomplete lineage sorting, which can distort phylogenetic inference. Indeed, recent studies have demonstrated that past mitochondrial introgression or incomplete lineage sorting can cause a severe discrepancy between mitochondrial and nuclear gene phylogenies, such as the misleading mitochondrial similarity observed between *Ixodes mojavensis* and *Ixodes minor* [14]. Furthermore, older techniques such as AFLP are dominant, exhibit low reproducibility and are inadequate for accurately assessing population heterozygosity. With the rapid development of high-throughput sequencing technologies, genome-wide approaches, such as restriction site-associated DNA sequencing (RAD-seq) and genotyping-by-sequencing (GBS), have become increasingly adopted in studies of tick population genomics. By generating thousands of single nucleotide polymorphisms (SNPs), these methods provide enhanced resolution for detecting fine-scale population structure and demographic history [15]. Nevertheless, genome-wide sequencing typically requires substantial financial investment, high-quality DNA and a laboratory with advanced bioinformatics capacity, which may constrain its implementation in long-term ecological monitoring or large-scale spatial surveillance.

Microsatellite (simple sequence repeat [SSR]) markers have been widely used in population genetic structure analyses due to their codominant inheritance, high polymorphism and good reproducibility. Due to their multi-allelic nature, highly polymorphic SSRs often provide greater information content per locus. Consequently, studies have demonstrated that to achieve comparable statistical power in estimating population structure parameters, it typically requires seven- to 11-fold as many biallelic SNPs to match the performance of a given set of microsatellites [16]. This high statistical efficiency makes SSRs particularly suitable for genetic discrimination of geographically isolated populations or closely related species [17]. In tick research, SSR markers have been successfully applied to elucidate the population genetic patterns of *Ixodes ricinus* in Europe [18] and the role of ticks in the epidemiology of African swine fever [19]. However, for *D. nuttalli*, an important tick species in northern China, there is still a lack of efficiently developed SSR markers and systematic population genetic studies.

To address this gap, we developed highly polymorphic SSR markers based on genome-wide data and applied them to investigate the genetic diversity and population structure analyses of *D. nuttalli* across northern China. This study provides new insights into the dispersal potential and evolutionary dynamics of *D. nuttalli*, and offers a theoretical foundation for regional monitoring and targeted control of tick-borne diseases.

## Methods

### Sample collection and DNA extraction

The adult *D. nuttalli* ticks analyzed in this study were collected from host animals at predefined sampling sites in northern China strictly during their peak period, from March 2023 to June 2024. The sampling followed a pre-designed approach based on the natural availability of ticks. Ticks were manually removed from hosts using fine-tipped forceps, ensuring minimal disturbance and avoiding injury to the animals. Once removed, ticks were immediately transferred into labeled cryovials, with all relevant collection metadata recorded. The collected specimens were placed in ventilated tubes and transported alive to the laboratory. Upon arrival, the live ticks were immediately identified morphologically.

Following identification, each tick was individually washed twice with 75% ethanol, rinsed once with ultra-pure water, air-dried and stored in a 1.5-ml centrifuge tube at − 80 °C until DNA extraction. Genomic DNA was extracted using the DNeasy Blood and Tissue Kit (QIAGEN, Hilden, Germany) according to the manufacturer's instructions. The quality and concentration of extracted DNA were assessed using a NanoDrop ND1000 spectrophotometer (Thermo Fisher Scientific, Waltham, MA, USA) and 1.5% agarose gel electrophoresis. Only samples meeting the following criteria were included in subsequent analysis: (i) ratio of absorbance at 260 nm and A280 nm (A260/A280) of between 1.8 and 2.0; (ii) ratio of absorbance at 260 nm and 230 nm (A260/A230) > 2.0; and (iii) intact electrophoretic bands without visible degradation.

### Identification of SSRs and primer design

The *D. nuttalli* reference genome (BioProject: PRJNA1255735) available in the National Center for Biotechnology Information (NCBI) database was used for SSR mining and primer design. Microsatellite loci were identified using MISA ( (MIcroSAtellite identification tool; http://pgrc.ipk-gatersleben.de/misa/misa.html) with the following criteria: minimum repeat units of 10, 6, 5, 5, 5 and 5 for mono- to hexanucleotides, respectively, and a minimum distance of 100 bp between adjacent SSRs. These relatively stringent minimum repeat thresholds were deliberately selected to target longer SSR tracts, as mutation rates and allelic polymorphism are positively correlated with repeat number; consequently, these thresholds enrich the candidate pool for highly informative markers. Default parameters were applied for the remaining settings. Primers were designed in batches using Primer 3 [20] with the following parameters: primer length 18–25 bp; annealing temperature (Tm) between 55 °C and 62 °C; GC content 40–60%; expected amplicon size 90–300 bp; and avoidance of primer–dimer and hairpin structures.

To obtain high-quality SSR markers suitable for population genetics, we adopted a multi-step screening strategy. Primers were initially stratified by repeat type (mono- to hexanucleotide). While limiting the inclusion of mononucleotide repeats, the remaining loci were sampled proportionally to reflect their natural genomic abundance. Specifically, the 70 candidate primer pairs selected for initial screening comprised 5 mono-, 30 di-, 20 tri-, 13 tetra-, 1 penta- and 1 hexa-nucleotide repeats. These 70 pairs were screened using DNA from 24 tick individuals to assess amplification efficiency and band clarity. Twenty-three primer pairs showing clear and stable amplification were selected for further sequencing validation. Polymorphism Information Content (PIC) was calculated, and markers were classified as highly polymorphic (PIC > 0.5), moderately polymorphic (0.25 < PIC ≤ 0.5) or lowly polymorphic (PIC ≤ 0.25) according to Botstein et al. [21]. Among the 23 successfully amplified primer pairs, 17 exhibited clear polymorphism and were prioritized for downstream analysis. To finalize the marker panel, these 17 loci were evaluated for null alleles using Micro-Checker software and tested for Hardy–Weinberg equilibrium (HWE) deviations. The explicit criterion for locus exclusion was the simultaneous presence of consistent null alleles and significant deviation from HWE across multiple populations after applying a Bonferroni correction.

### SSR genotyping

Simple sequence repeat loci with stable amplification and high discriminative power were selected for genotyping. Forward primers were labeled with the fluorescent dye 6-FAM. PCR amplification was performed in a 25-μl reaction volume containing 0.5 μl template DNA, 1 μl of each primer, 12.5 μl of 2 × EasyTaq PCR SuperMix (TransGen, Beijing, China) and 11 μl ddH₂O. Reactions were performed on a Veriti 96 Well Thermal Cycler (Applied Biosystems Asia Pte. Ltd., Singapore) under the following conditions: initial denaturation at 94 °C for 5 min; 35 cycles of 94 °C for 30 s; annealing at locus-specific optimized temperatures ranging from 53 °C to 60 °C for 30 s and extension at 72 °C for 60 s; followed by a final extension at 72 °C for 5 min. PCR products were visualized via 1.5% agarose gel electrophoresis using DL500 (NimaGen, Nijmegen, The Netherlands) as a size standard. Amplicons with expected sizes were sent to Tsingke Biotechnology Co., Ltd. (Beijing, China) for capillary electrophoresis, using GeneScan 500 LIZ (Thermo Fisher Scientific) as the internal size standard. Allele calling and fragment sizing were performed using GeneMarker V2.2.0 (SoftGenetics, State College, PA, USA).

### Data analysis

Potential null alleles were assessed using MICRO-CHECKER v2.2.3 [22], as the presence of null alleles may affect estimates of genetic diversity and population structure [23, 24]. Deviations from HWE for each locus–population combination were tested in GenePop [25], with significance levels adjusted via Bonferroni correction (*α* = 0.05) [26]. Loci were excluded from further analyses if MICRO-CHECKER indicated the presence of null alleles and GenePop showed significant HWE deviation in at least two of the five sampled populations.

Genetic diversity parameters—including the number of alleles (*N*a), effective number of alleles (*N*e), Shannon’s information index (SI), observed heterozygosity (*H*o) and expected heterozygosity (*H*e)—were calculated using GenAlEx v6.5 [27]. Nei’s genetic distance (*D*) and identity (*I*) were also estimated. The PIC for each locus was computed in PowerMarker v3.25 [28] using the formula:

$$PIC=1-{\sum}_{i=1}^{n}{p}_{i}^{2}$$

where *n* represents the total number of alleles at a given locus and *p*_*i*_ represents the frequency of the *i*th allele [29].

Population structure was inferred using a Bayesian clustering approach implemented in the STRUCTURE v2.3.4 software package [30]. The admixture model with correlated allele frequencies was applied, as this model is appropriate for populations that may share ancestry or experience gene flow. No prior information on sampling locations (i.e. the LOCPRIOR option was not used) was incorporated into the analysis, and clustering was based solely on multi-locus genotype data. The number of clusters (*K*) ranged from two to five, corresponding to the number of sampled geographic populations (*n* = 5), thereby allowing the possibility that each sampling locality represented a distinct genetic cluster. For each *K*, 15 independent replicate runs were performed, each with a burn-in of 10,000 iterations followed by 100,000 Markov chain Monte Carlo (MCMC) iterations. The optimal *K* value was determined using the Δ*K* method, and results were summarized and visualized using StructureSelector software (https://lmme.ac.cn/StructureSelector/).

Principal coordinate analysis (PCoA) was conducted in GenAlEx using codominant genotypic distances to visualize genetic relationships among and within populations. A phylogenetic tree was constructed based on Nei’s genetic distance using MEGA 11 [31] and visualized in the Interactive Tree Of Life (ITOL) tool [32]. Pairwise population differentiation (F_ST_ [fixation index]) and analysis of molecular variance (AMOVA) were performed in Arlequin 3.5 [33]. The degree of genetic differentiation was interpreted based on standard qualitative guidelines: F_ST_ < 0.05 indicates little differentiation; 0.05–0.15 indicates moderate differentiation; 0.15–0.25 indicates great differentiation; and F_ST_ > 0.25 indicates very great differentiation [34]. Additionally, values of F_ST_ > 0.15 were considered to be indicative of high differentiation [35]. A Mantel test was carried out in GenAlEx (Genetic Analysis in Excel) software to evaluate the correlation between F_ST_ and geographic distance (km).

## Results

### Sample information

A total of 192 *D. nuttalli* ticks were collected from five locations in northern China: Hohhot (HH), Hulunbuir (HB), Bayannur (BN), Ulanqab (WC) and Yan’an (SX) (Fig. 2a). Except for the Hohhot samples, which were collected from Amur hedgehogs (*Erinaceus amurensis*), all other specimens were obtained from domestic sheep (*Ovis aries*). Host populations inhabited a range of open habitats, including grasslands, pastures and shrublands. Specific demographic information regarding the geographic distribution of sampling sites and the sample size obtained from each site are provided in Table 1.

**Table 1:** Detailed information on *Dermacentor nuttalli* populations in northern China

Population ID	Full name of sampling sites	Parasitic status	Host species	Habitat type	Sample size	Latitude (°N)	Longitude (°E)
SX	Yan’an Shaanxi	Parasitic	*Ovis aries*	Pasture	26	36.7252	107.9158
HH	Hohhot, Inner Mongolia	Parasitic	*Erinaceus amurensis*	Shrubland	56	40.8333	111.0213
HB	Hulunbuir Inner Mongolia	Parasitic	*Ovis aries*	Grassland	48	48.1311	116.2671
BN	Bayannur Inner Mongolia	Parasitic	*Ovis aries*	Pasture	14	40.7418	107.0749
WC	Ulanqab Inner Mongolia	Parasitic	*Ovis aries*	Pasture	48	41.1808	112.4844

### Characterization of developed SSR markers

**Fig. 1:**
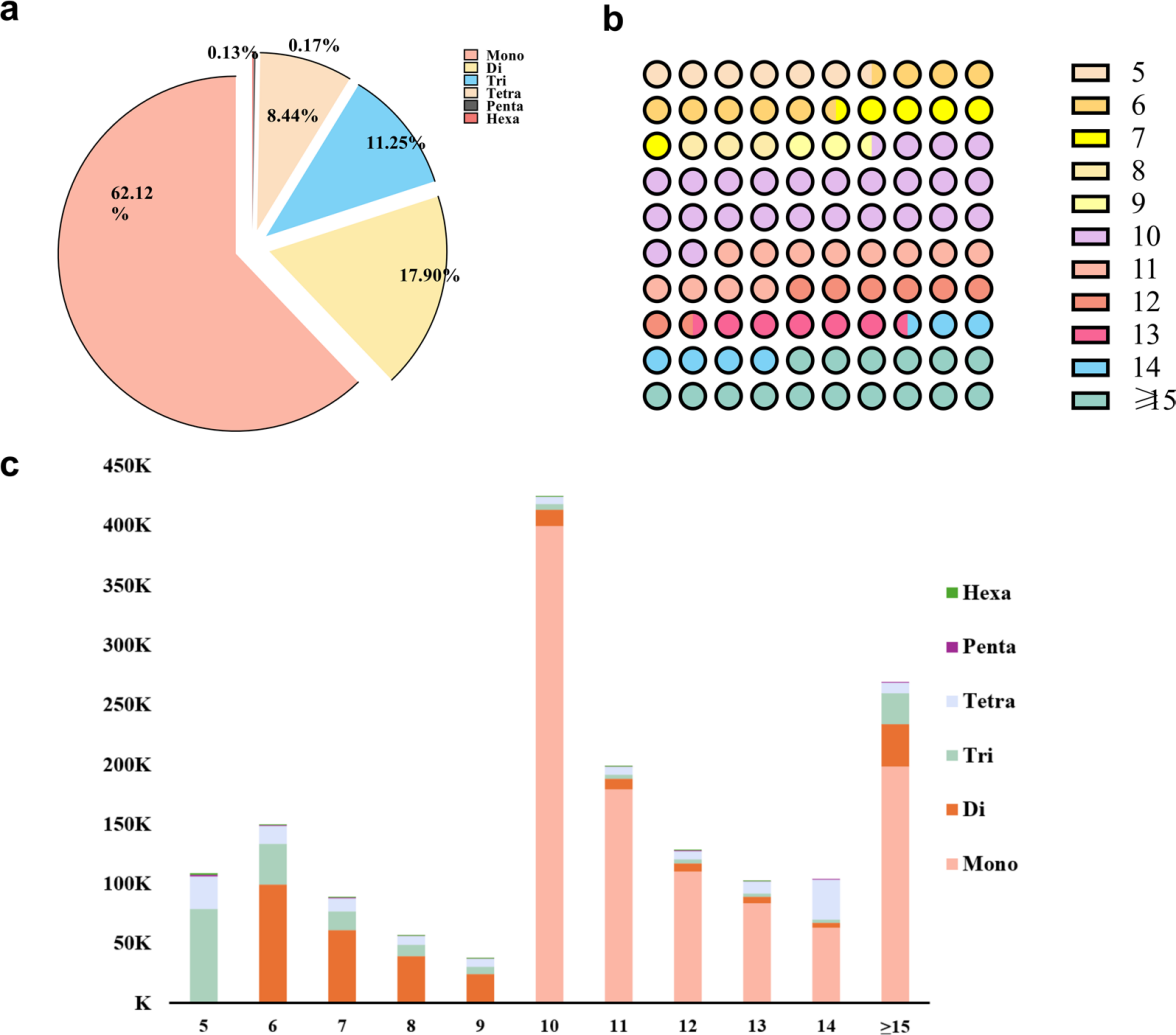
Characteristics of simple sequence repeat (SSR) motifs in *Dermacentor nuttalli*. **a** Distribution of different SSR motif types. **b** Frequency distribution of SSRs by number of repeat units. **c** Relationship between motif type and repetition frequency

A genome-wide scan using MISA identified 1,665,824 SSR loci (see Additional file 1: Table S1 for detailed summary statistics of all identified markers). Among the six SSR types, mononucleotide repeats were the most abundant (1,034,744; 62.1%), followed by di- (298,188; 17.9%), tri- (187,421; 11.3%), tetra- (140,628; 8.4%), penta- (2,758; 0.2%) and hexanucleotide (2,085; 0.1%) repeats (Fig. 1a). Tandem repeat numbers ranged from five to 63, with the majority (77.6%) falling in the range of five to 13 repeats (Fig. 1b, c). Among the mononucleotide repeats, A/T motifs (924,253; 89.3%) were predominant compared to C/G motifs. For dinucleotide repeats, AG/CT (51.8%) was the most frequent motif, followed by AC/GT (38.0%) and AT/AT (5.5%). The most common tri- and tetranucleotide motifs were ATC/ATG (47.2%) and AATC/ATTG (12.5%), respectively. Penta- and hexanucleotide repeats were rare, with the dominant motifs being AACCT/AGGTT (13.6%) and ACACGC/CGTGTG (55.4%) (see Additional file 2: Table S2 for the distribution and abundance of the 20 most frequent SSR motif types).

### Genetic diversity within populations

Following the established screening criteria, we assessed the 17 polymorphic loci amplified by these primer pairs using Micro-Checker analysis and HWE testing. Two loci were excluded because they consistently exhibited both a high frequency of null alleles and significant deviations from HWE across multiple populations after Bonferroni correction. Ultimately, 15 highly reliable SSR markers were retained for the final genetic analysis (see Additional file 3: Table S3 for the specific primer sequences and properties of these developed loci).

Across all 192 individuals, a total of 83 alleles were detected across the 15 loci, with an mean number of alleles per locus of 5.52 (Table 2). The *N*e ranged from 1.26 to 6.37 (mean 2.61); the SI varied between 0.27 and 1.45 (mean 0.91); *H*o ranged from 0.03 to 0.57 (mean 0.14); and* H*e ranged from 0.15 to 0.78 (mean 0.44). The mean PIC was 0.66, with three loci showing moderate polymorphism (0.25 < PIC < 0.5; namely loci 666657, 1708630 and 539540) and the remaining 12 loci exhibiting high polymorphism (PIC > 0.5). Based on the thresholds defined by Hartl and Clark [34] and Frankham et al. [35], the F_ST_ across loci was 0.34, with three showing moderate differentiation (0.05 < F_ST_ < 0.15; namely loci 763105, 49363 and 539540) and the remaining 12 loci showing high differentiation (F_ST_ ≥ 0.15).

**Table 2:** Genetic diversity indices of the 15 simple sequence repeat loci in *Dermacentor nuttalli*

Locus	Motif	*N*a	*N*e	SI	*H*o	*H*e	PIC	F_ST_	HWE
476372	(TTCT)10	12.00	6.37	1.26	0.26	0.44	0.77	0.33	ns
666657	(TTA)5	2.80	1.26	0.28	0.03	0.15	0.48	0.60	ns
763105	(A)13	5.60	2.47	1.06	0.57	0.54	0.57	0.10	ns
9853	(T)11	8.60	2.90	1.14	0.14	0.48	0.74	0.30	ns
49363	(T)15	6.60	3.61	1.37	0.17	0.65	0.71	0.14	ns
1396081	(A)12	6.20	3.71	1.45	0.08	0.71	0.84	0.15	ns
125766	(GGA)5	3.20	1.54	0.52	0.07	0.28	0.59	0.56	ns
3955559	(CGT)5	4.00	1.65	0.61	0.11	0.32	0.56	0.46	ns
2007637	(GAT)5	8.00	2.91	1.32	0.10	0.61	0.76	0.22	ns
1708630	(AAT)5	2.20	1.37	0.27	0.04	0.15	0.40	0.51	ns
878212	(CCG)6	4.40	1.58	0.61	0.09	0.29	0.77	0.62	ns
539540	(CTC)5	3.20	1.48	0.52	0.12	0.29	0.36	0.14	ns
343878	(GCC)6	5.40	3.19	1.30	0.13	0.67	0.84	0.22	ns
1586794	(GTT)5	5.80	2.81	1.18	0.12	0.63	0.82	0.24	ns
167118	(AAAATG)5	4.80	2.27	0.74	0.08	0.34	0.71	0.47	ns
Mean		5.52	2.61	0.91	0.14	0.44	0.67	0.34	

*F*_*ST*_ Pairwise population differentiation, *He* expected heterozygosity, *Ho* observed heterozygosity, *HWE*, Hardy–Weinberg equilibrium, *Na* mean number of alleles per locus across populations, *Ne* number of effective alleles, *ns* not significant, *PIC* polymorphism information content

*P < 0.05, **P < 0.01, *** *P* < 0.001

At the population level, genetic diversity indices varied considerably (Table 3). The *N*a per population ranged from 2.87 to 10.00; *N*e, from 1.89 to 4.50; SI, from 0.63 to 1.39; *H*o, from 0.09 to 0.19; *H*e from 0.34 to 0.58; and the PIC, from 0.31 to 0.57. Three populations showed moderate genetic diversity (PIC > 0.25), while the HH and HB populations exhibited high diversity (PIC > 0.50). The HB population displayed the highest values for *N*e (4.50), SI (1.36), *H*o (0.19), *H*e (0.58) and PIC (0.57), suggesting a consistently large effective population size, historical demographic stability or extensive gene flow within this region. Conversely, the BN population generally exhibited the lowest diversity metrics (e.g. *N*a = 2.867, PIC = 0.311). However, it is important to note that the BN population also possessed a relatively small sample size (*n* = 14), which may have affected the precision of these specific diversity estimates.

**Table 3:** Genetic diversity indices of five populations of *Dermacentor nuttalli*

Population^a^	*Na*	*Ne*	SI	*H*o	*H*e	PIC
SX	4.000	1.927	0.684	0.123	0.342	0.317
HH	5.867	2.573	1.056	0.155	0.539	0.505
HB	10.000	4.495	1.385	0.189	0.576	0.570
BN	2.867	1.886	0.629	0.138	0.342	0.311
WC	4.867	2.159	0.792	0.086	0.382	0.365
Mean	5.520	2.608	0.909	0.138	0.436	0.414

*He* Expected heterozygosity, *Ho* observed heterozygosity, *Na* mean number of alleles per locus across populations, *Ne* number of effective alleles, *PIC* polymorphism information content* SI* Shannon’s information index

^a^See Table 1 for full name of population and geographic location

### Population genetic structure

**Fig. 2:**
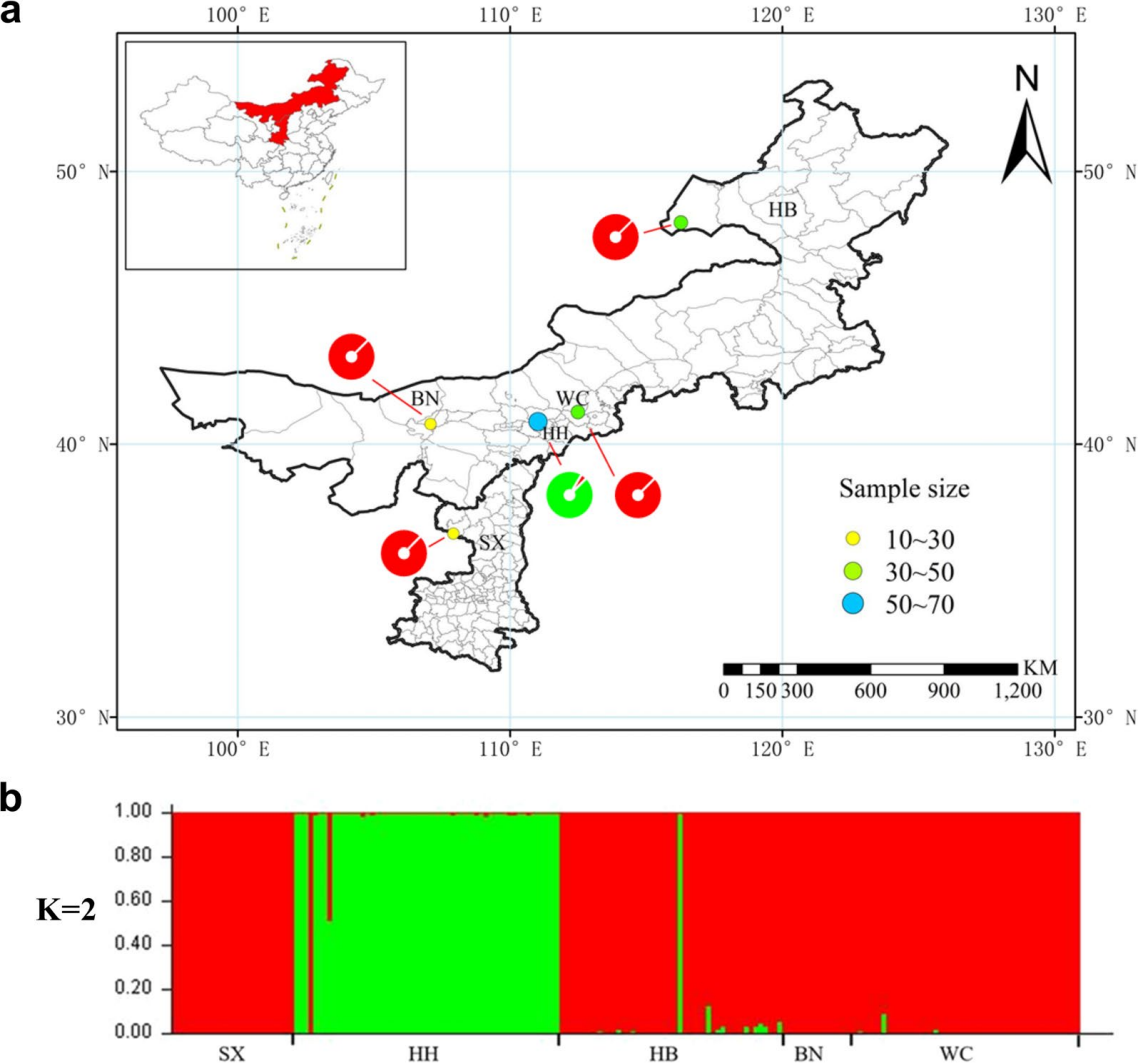
Population genetic structure of *Dermacentor nuttalli* based on STRUCTURE analysis (5 populations, number of individuals [* n*] = 192). **a**, Geographic distribution of the five populations, with pie charts representing the proportional membership (Q) to the two genetic clusters (*K* = 2). **b**, Bar plot output from STRUCTURE (*K* = 2), where each vertical bar represents an individual, and colors denote the estimated ancestry proportion to each cluster. BN, Bayannur; HB Hulunbuir; HH, Hohot; *K*, number of genetic clusters, SX, Yan'an; WC, Ulanqab

Bayesian clustering analysis in STRUCTURE revealed the optimal number of genetic clusters as *K* = 2 (Δ*K* method; Additional file 4: Figure S1). The five populations were divided into two groups: one group included SX, HB, BN and WC (red cluster), and the other consisted solely of HH (green cluster) (Fig. 2a). Specifically, all except one admixed individual from the HH population exhibited a membership coefficient > 0.75 for the green cluster, indicating predominantly pure genetic ancestry within these groups (Fig. 2b).

**Fig. 3:**
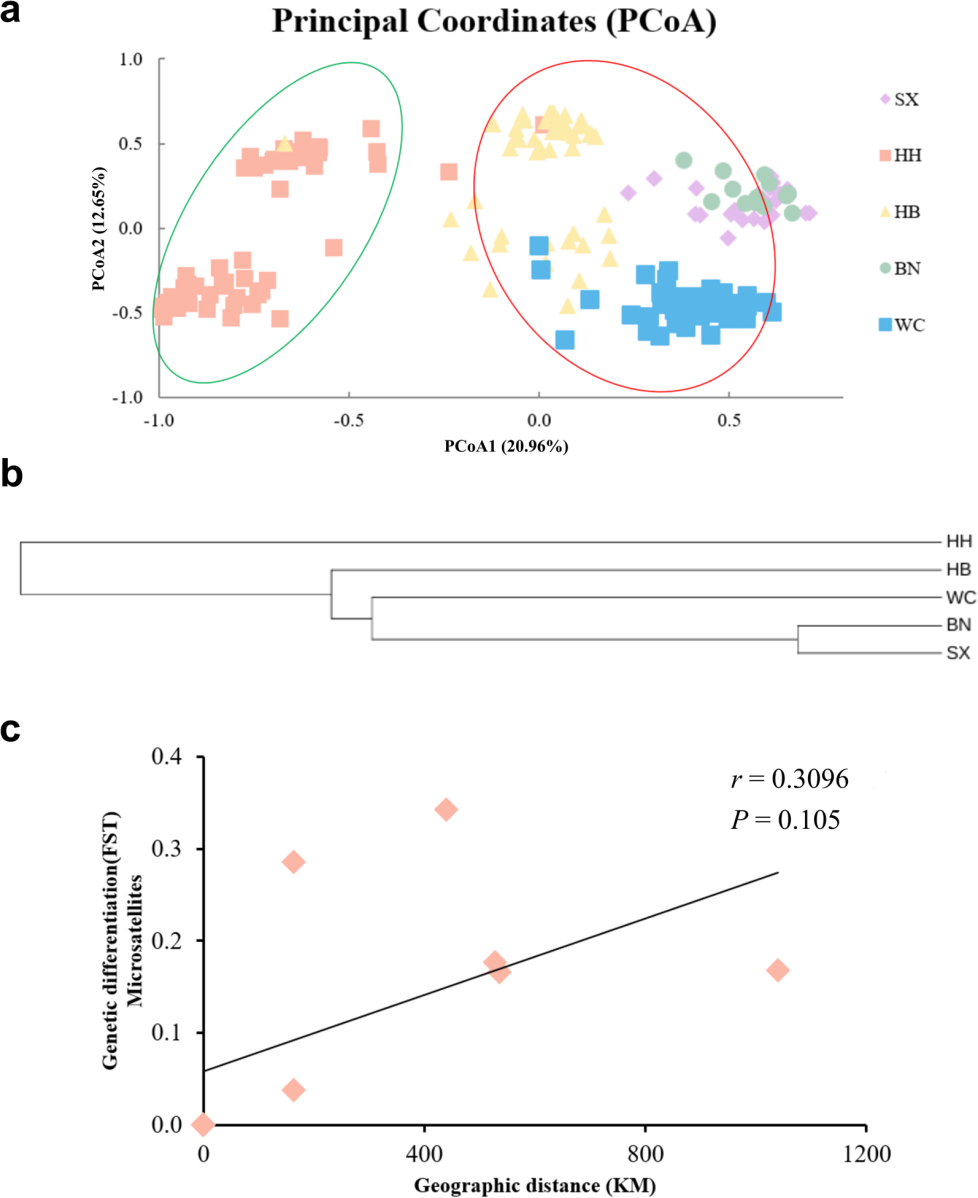
Genetic analysis of five *Dermacentor nuttalli* populations using 15 SSR loci. **a**, The PCoA of 192 individuals. **b**, Phylogenetic tree constructed from Nei’s unbiased genetic distance. **c**, Isolation-by-distance pattern assessed by linear regression of pairwise population F_ST_ values against geographical distance. BN, Bayannur; F_ST_, pairwise population differentiation; HB, Hulunbuir; HH, Hohot; *K*, number of genetic clusters; PCoA, principal coordinate analysis; SX, Yan'an; WC, Ulanqab

The PCoA also clearly separated HH from the other four populations (Fig. 3a), consistent with the UPGMA (unweighted pair group method with arithmetic mean) dendrogram (Fig. 3b), which grouped SX, BN, WC and HB, with SX and BN being the closest. A Mantel test revealed a weak positive correlation between genetic distance (F_ST_) and geographic distance (*r* = 0.31, *P* = 0.11; Fig. 3c), indicating that isolation-by-distance was not statistically significant. This suggests that genetic differentiation may be influenced by factors beyond geography, such as host-mediated dispersal or anthropogenic activities.

### Population differentiation and genetic variation

The AMOVA indicated that 36.9% of the total genetic variation occurred among populations, while 63.1% was within populations (Table 4), reflecting substantial intra-population diversity. The overall high genetic differentiation (F_ST_ = 0.37) suggests limited gene flow, likely due to geographical and ecological barriers. Pairwise F_ST_ values and gene flow (Nm) estimates between populations are shown in Table 5. Notably, the HH population exhibited consistently higher genetic differentiation when compared to all other populations than did the remaining populations among themselves, further supporting its distinct genetic isolation. For example, the lowest genetic differentiation was between BN and SX (F_ST_ = 0.04; Nm = 6.33), indicating frequent gene exchange. In contrast, the highest differentiation was between BN and HH (F_ST_ = 0.34; Nm = 0.48), suggesting strongly restricted gene flow.

**Table 4:** Analysis of molecular variance from five *Dermacentor nuttalli* populations

Source of variation	*df*	SS	Variance components	Percentage of variation
Among populations	4	612.00	2.03 Va	36.9%
Within populations	379	1317.69	3.48 Vb	63.1%
Total	383	1929.69	5.51	100.0%

*df* Degrees of freedom, *SS*, sum of squares

**Table 5:** Genetic differentiation coefficient (F_ST_, below diagonal) and gene flow (Nm, above diagonal) among five *D. nuttalli* populations

Populations^a^	SX	HH	HB	BN	WC
SX	0.00	0.50	1.24	6.33	1.26
HH	0.33	0.00	1.11	0.48	0.62
HB	0.17	0.18	0.00	1.23	1.17
BN	0.04	0.34	0.17	0.00	1.17
WC	0.17	0.29	0.18	0.18	0.00

^a^See Table 1 for full name of population and geographic location

## Discussion

This study developed the first panel of genomic microsatellite markers for *D. nuttalli* and applied them to evaluate genetic diversity and population structure across five geographic populations in northern China. The results revealed moderate to high genetic diversity and a high and statistically significant level of population differentiation (mean F_ST_ = 0.37), with two distinct genetic clusters identified. Notably, genetic differentiation showed no significant association with geographic distance, suggesting that non-geographic factors—such as host-mediated dispersal and anthropogenic activities—play predominant roles in shaping the population structure of this tick species.

A large number of putative SSR loci were identified from the *D. nuttalli* genome. Given that ixodid ticks possess large and highly repetitive genomes (2.0 – 3.0 Gb), the observed abundance of SSRs likely reflects genome size rather than unusual microsatellite enrichment [36]. The validated microsatellite markers exhibited high levels of polymorphism (mean PIC = 0.66), indicating that they are informative for assessing genetic variation within *D. nuttalli*. Consistent with the general characteristics of SSRs, these markers are multi-allelic and codominant, enabling reliable estimation of heterozygosity and population structure [17, 37]. Although genome-wide SNPs are increasingly being adopted, SSRs retain practical advantages in certain research contexts due to their multi-allelic and codominant properties. Because each SSR locus can harbor multiple alleles, a moderate number of well-characterized loci may provide substantial discriminatory power for population-level analyses. In addition, SSR-based approaches generally require lower sequencing depth and more modest bioinformatic resources, making them suitable for large-scale ecological surveys or routine monitoring programs. The marker panel developed in this study exhibited considerable allele richness and strong informativeness, supporting its utility for future population genetic and epidemiological investigations of *D. nuttalli*.

The *H*e values across the five populations (0.34–0.58) were generally higher than the *H*o values and lower than those reported for *Rhipicephalus appendiculatus* (*H*e = 0.63–0.67) [38], but within the range for *Dermacentor reticulatus* (*H*e = 0.36–0.60) [39], indicating moderate genetic diversity. The consistently lower *H*o than *H*e values across populations may reflect heterozygote deficiency, which is likely associated with limited sampling coverage or undetected local substructure within these broad geographic sampling regions [18]. Among the populations, HB showed the highest genetic diversity (*N*e = 4.50, *H*e = 0.58, PIC = 0.57), which may be attributed to the region’s relatively intact grassland ecosystem, diverse host resources (including abundant rodents and herbivores) [8], as well as livestock grazing, tourism and cross-border transportation that facilitate tick dispersal and gene flow.

The clear divergence into two genetic clusters—HH versus the other four populations—was consistently supported by the STRUCTURE, PCoA, and UPGMA analyses. The AMOVA revealed that 36.89% of genetic variation occurred among populations, with 63.11% within populations, indicating a high and statistically significant level of genetic differentiation (F_ST_ = 0.37). The F_ST_ values ranged from 0.04 to 0.34, and Nm estimates varied widely (0.48–6.33). For example, the lowest differentiation and highest gene flow were observed between BN and SX (F_ST_ = 0.04, Nm = 6.33), whereas the highest differentiation and lowest gene flow were found between BN and HH (F_ST_ = 0.34, Nm = 0.48). The Mantel test revealed a weak, non-significant positive correlation between genetic and geographic distances (*r* = 0.31, *P* = 0.11). It should be noted that this Mantel test was based on only 10 pairwise comparisons among the five sampled populations. While this limited number of comparisons inherently reduces the statistical power to detect subtle spatial gradients, the absence of a strong linear relationship is consistent with our biological expectations. These results suggest that factors other than simple geographic distance—such as host movement, ecological barriers or human-mediated translocation—are likely contributing to genetic differentiation among populations. Similar patterns have been reported in other tick species, such as *Dermacentor andersoni* [12] and *Amblyomma maculatum* [40], in which genetic structure was influenced by host-mediated dispersal and anthropogenic factors. Epidemiologically, these findings support targeted control strategies. For example, the genetically isolated HH cluster—collected exclusively from wild hedgehogs (*E. amurensis*) in shrublands—likely represents a distinct, wildlife-maintained transmission cycle for tick-borne pathogens. This contrasts sharply with the interconnected populations collected from livestock (*O. aries*) in pastures. Consequently, vector control must be customized: interventions in shrubland habitats should prioritize wildlife monitoring, whereas strategies in pasture regions should focus on livestock management and movement restrictions.

This study provides new insights into the population genetic characteristics of *D. nuttalli*; however, several limitations to the study should be acknowledged. First, the geographic scope of sampling was restricted to five localities within a single broader region and therefore may not capture the full extent of genetic diversity across the species’ wider distribution. Second, sample collection was based on naturally occurring infestations in available hosts at each site, resulting in unequal numbers of specimens among localities. Although such variation reflects field conditions, it may have influenced the precision of comparisons among populations. Third, to reduce potential temporal heterogeneity, all specimens included in the genetic analyses were obtained during a narrow sampling window from April to May. This design likely minimized, but could not entirely exclude, temporal effects on genetic structure. Fourth, the use of nuclear SSR markers alone constrained inference regarding deeper phylogeographic history, maternal lineage structure and signatures of fine-scale adaptive variation. Future studies would benefit from integrating complementary marker systems, including mitochondrial loci such as *cox1* or 16S ribosomal RNA to investigate maternal lineages and historical demography, together with genome-wide SNP data generated through reduced-representation sequencing approaches, such as RAD-seq or GBS, to improve genomic resolution. Broader sampling across geographic regions, host species and seasons, combined with ecological and epidemiological data, will be important for clarifying the drivers of population structure and their potential relevance to pathogen transmission dynamics.

## Conclusions

This study successfully developed a set of polymorphic SSR markers for *D. nuttalli* and applied them to investigate the genetic structure of its populations in northern China. The results revealed moderate genetic differentiation among geographic populations and suggested that host movement, human activities and ecological connectivity may play a greater role in shaping genetic structure than geographic distance alone. The 15-locus SSR panel developed here represents a transferable genetic resource that can be readily applied in future studies across a broader range of *D. nuttalli* populations and ecological contexts. These findings offer valuable genetic insights into the dispersal dynamics and evolutionary history of *D. nuttalli*, thereby establishing a solid foundation for future investigations into ecological adaptability and pathogen transmission risks.

## Supplementary Information

**Additional file 1: Table S1. **Summary statistics of SSR markers identified in the *D. nuttalli* genome.

**Additional file 2: Table S2.** Distribution and abundance of the 20 most frequent SSR motif types in *D. nuttalli*.

**Additional file 3: Table S3.** Primer sequences and properties of 15 developed SSR loci for *D. nuttalli*.

**Additional file 4: Figure S1.** Estimation of the optimal genetic cluster number (*K*) using Delta* K* statistics.

## Data Availability

The datasets supporting the conclusions of this article are included within the article and its supplementary files. The* Dermacentor nuttalli *reference genome used in this study is available in the NCBI database under BioProject PRJNA1255735.
