# Supplementary figures and images for: Genetic diversity and population structure of the Dermacentor nuttalli in Northern China inferred from microsatellite markers
Source: Parasit Vectors. 2026 May 7;19:265. doi: 10.1186/s13071-026-07426-w (PMC13317360; doi:10.1186/s13071-026-07426-w)

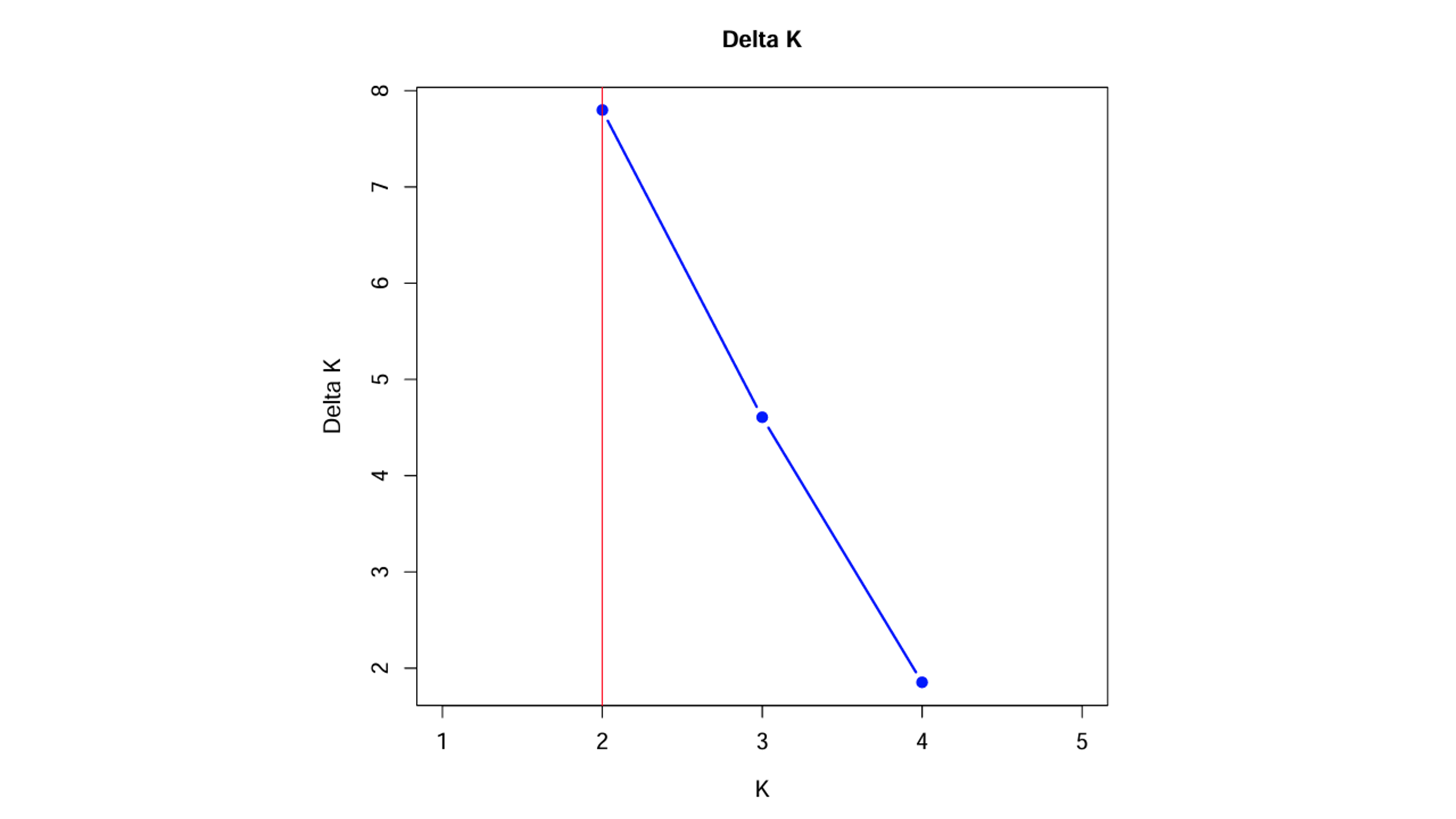

Supplement: Supplementary file 4 — Additional file 4: Figure S1. Estimation of the optimal genetic cluster number (K) using Delta K statistics. [file 13071_2026_7426_MOESM4_ESM.tif]
